# Dr. Denman, on a Remarkable Abscess Formed after Child-Birth

**Published:** 1799-08

**Authors:** Thomas Denman


### *To the Editors of the Medical and Phyſical Journal.*

GENTLEMEN,

IF you think the hiſtory of the following caſes not unſuitable to the deſign of your very uſeful publication, I beg the favour of you to inſert it.

I am, Gentlemen,

Your very humble Servant,

*July 6th*, 1799. THOMAS DENMAN.

CASE I.—In July, 1798, I was deſired to viſit a lady, of whoſe caſe I received this account:—

On June the 10th, ſhe had been delivered of a dead child, between the ſeventh and eighth month of her pregnancy, when ſhe ſuffered very acute pain in the extraction of the *placenta*, which was thought neceſſary. For ſeveral days previous to her delivery ſhe had a conſiderable degree of fever, and much general uneaſineſs over the *abdomen*, for which ſhe was bled, and took ſome cooling and quieting medicines. On the 12th (the ſecond day after her delivery), ſhe had a ſtrong and violent rigor, ſucceeded by very ſevere pain in her left ſide, near the ſpine of the *ilium*, and fever, which continued for ſeveral days, when her milk (before ſecreted) entirely diſappeared.

Though the pain and fever were abated, they never entirely left her; and after another rigor on the 19th, with an increaſe of fever and pain in the part firſt affected, her friends were alarmed, and a phyſician of eminence was deſired to ſee her. He preſcribed what the ſituation and circumſtances of the patient ſeemed to require, and ſhe was much relieved. There were, however, frequent exacerbations of fever; the pain of which ſhe originally complained never entirely left her, and was ſometimes violent. It was now perceived ſhe had no power of moving her left leg or thigh, and ſhe herſelf was ſenſible of a deep-ſeated ſwelling on the left ſide of the *abdomen*, though it could not be diſcovered by her attendants. A bliſter was applied to the whole of the pained ſide, and after ſome days farther attendance, the phyſician withdrew, recommending her to go into the country, and encouraging her to hope that, as ſhe recovered her ſtrength, her complaints would leave her. She was alſo adviſed to uſe as much exerciſe as ſhe could, and accordingly attempted every day to walk with a crutch, and the help of her nurſe; but every attempt gave her excruciating pain, and ſhe was daily ſenſible of loſing, inſtead of gaining ſtrength.

I firſt ſaw her on the 28th of July. As there was an evident fulneſs on the left ſide of the *abdomen*, with much pain on preſſure, loſs of appetite, and other ſymptoms of fever, from ſome degree of which ſhe was, in fact, never entirely free, I directed three or four leeches to be applied to the part affected, and to be repeated every other day, and ſuch medicines as were likely to abate the fever, to keep the bowels gently open, and to moderate the pain. She was ſomewhat relieved by theſe means, and as ſhe was very weak, I tried the *bark*, and ſome other tonic medicines, from which ſhe did not apparently receive any benefit. From the contraction and waſting of the limb, and from the other circumſtances before recited, thinking it probable that an abſceſs had begun to be formed in ſome part of the cavity of the *abdomen*, I requeſted to have a conſultation, and Dr. Baillie was called in. After a mature deliberation on all the preceding circumſtances, and the preſent ſtate of the patient, it ſeemed moſt reaſonable to think that an abſceſs was forming in the *pſoas* muſcle. Small doſes of *cicuta* in the ſaline draughts were preſcribed, and a ſoft plaiſter with *opium* was applied to the ſide; the caſe of the patient ſeeming to admit of little other relief than ſome alleviation of her ſuffering. In the middle of *Auguſt* ſhe returned to her houſe in town, not in any reſpect amended in her general health, and ſhe ſuffered more from her local complaints.

In a few days after her arrival in town, the pain being much increaſed, ſhe went into the warm bath, and on the following day ſhe was ſuddenly relieved by diſcharging a very large quantity of purulent matter, mixed with her urine. This was conſidered as a proof that an abſceſs had been formed, and diſcharged into the bladder, probably by means of an adheſion which had taken place, and a ſubſequent communication between this and the part firſt affected.

She continued to go into the warm bath for a few days, but ſuſpecting that ſhe was weakened, and feeling herſelf very much fatigued by it, ſhe relinquiſhed it altogether. At this time her medicines were changed for ſome of the milder turpentines, in ſmall doſes, and ſtill ſuffering conſiderable pain, opiates were given, and repeated as the caſe required.

When there was the greateſt quantity of purulent matter diſcharged with the urine, and ſometimes I think there could not have been leſs than four ounces at a ſingle evacuation, ſhe ſuffered the leaſt pain; but when there was a ſuſpenſion of the diſcharge, the pain was always moſt ſevere.

In the beginning of September, a ſwelling of a conſiderable ſize, with an evident fluctuation in it, was diſcovered on the inſide of the thigh, without any appearance of inflammation or redneſs of the ſkin, as if the fluctuating matter matter had been formed there; and, by a careful examination, the courſe by which the fluid had deſcended from the groin to the thigh, could be readily traced. The ſwelling gradually deſcended till it came very near the ham, varying in ſize, according to the poſition of the limb and body, and the patient thought ſhe could diſtinctly perceive both the deſcent and riſe of the fluid.

The night-ſweats, and other hectic ſymptoms, were now extreme; but, after a trial of the bark, and other medicines of that claſs, which diſagreed, ſhe for many weeks took no medicine whatever, except ſmall doſes of *opium*, when the pain was violent, and ſome gentle laxatives when ſhe was coſtive. She was allowed to drink porter at her meals, and at any other time, without reſtraint, when ſhe wiſhed for it, and always conſidered herſelf not only ſupported, but very much refreſhed by its uſe.

In October ſhe kept her bed altogether, unable to move, or help herſelf in any poſition, and frequently ſuffering much pain. I then propoſed a conſultation with Mr. Cline, the ſurgeon of the family, to conſider the propriety or expediency of making an opening in the tumour in the thigh, and by giving it an inferior vent, to prevent the matter from returning into the *abdomen.* Mr. Cline did not then think it juſtifiable to make an opening in the tumour, and I readily acquieſced in his opinion.

At the latter end of this month, ſhe was reduced to a ſtate of extreme weakneſs, and exceedingly emaciated, but her appetite, which had never entirely left her, now began to improve. The tumour in the thigh daily leſſened, and ſoon diſappeared altogether; as did the quantity of matter diſcharged with the urine, till that alſo entirely ceaſed. In November ſhe frequently voided ſmall quantities of blood with her ſtools, and at the latter end of that month her health and ſtrength were conſiderably improved. There was alſo about this time a return of ſome power of moving her limb; ſhe ſoon became able to walk with crutches, the infirm leg being ſupported in a ſtirrup; and ſhe had a return of the *menſes*, which had not before appeared ſince the time of her delivery.

On the 20th of December ſhe was lifted into the coach for the benefit of taking the air, and her health might at this time be ſaid to be reſtored, as ſhe had no complaint, and though weak and emaciated, was every day ſenſible of amendment.

In the beginning of the year ſhe again proved with child, and went on to the full period of pregnancy, when ſhe was ſafely delivered of a healthy boy; having recovered before the time of her delivery the perfect uſe of her her limb. She now walks, and performs all the offices of life with her accustomed ease, and has not the least remaining token of the complaint from which she had so severely suffered.

---

CASE II.—The following statement of the case of a lady, was given me by Mr. *Thomas*, a very respectable surgeon at *Tunbridge Wells*; who had attended her from the commencement of her illness.

This lady, who had had several children, was brought to bed in *January*, 1798; and had perfectly recovered her health. She menstruated regularly till the following *June*, when she became sensible of a pain in the right side of the *abdomen*, near the groin, which, though not violent, prevented her from lying with ease, or sleeping on that side. About the middle of *January*, 1799, she was suddenly seized with a violent pain in her bowels, tension of the *abdomen*, and much soreness on pressure, accompanied with vomiting, constipation, and frequent faintings. These complaints were relieved chiefly by glysters and gently purgative medicines, but not entirely removed without many repetitions of them. Before this attack, she had been much weakened by profuse discharges of blood from the *uterus*, and about ten days after, she suffered very violent pain in the lowest part of the back, seemingly near the extremity of the *sacrum*, which joins the *os coccygis*, extending to the loins and across to the hips, especially the right, and down that thigh. The slightest pressure on the *sacrum*, or hip, brought on excruciating pain in all the neighbouring parts, which continued for several minutes after the pressure was removed. This pain was considered as the *sciatica*, and it was relieved by the warm bath, and the occasional use of opiates. By a return of uterine hemorrhage, every six or eight days, together with loss of appetite and want of rest, she became extremely weak, irritable, and emaciated. On every return of uterine hemorrhage, the pains in the back were much encreased, as they also were by the evacuation of a costive stool, for which reason glysters were daily injected. She never had much difficulty in voiding her urine, but frequent inclination to do it; yet there never was in it any distempered appearance.

About the middle of *February*, she could bear to be turned from her back to her side, but at those times she felt as if some heavy substance was contained in the *abdomen*, which shifted its place as she was turned. After a confinement of six weeks to her bed, the painful symptoms were mitigated, she was able to sit in a chair, with her feet raised high and her knees drawn up, but she was soon obliged by the pain in her back, to return to a recumbent position; nor was she able to suffer her right leg to approach the ground or bear the least weight upon it.

Her

Her health and ftrength however gradually improved, and in *March* fhe was able to move and walk a little, but inftead of her former complaints, there was great tenfion and pain above the *offa pubis*, and the whole hypogaftric region was full and hard, but not fore to the touch, except on the right fide, where the hardnefs was firft perceived. One day about this time, while fhe was in the warm-bath, fhe difcovered a large and hard tumour, extending to the right fide of the navel, the increafe of which was fo rapid, that in the courfe of a few days it occupied the whole *abdomen*. She was then freed from pain in all the parts contained in the *pelvis*, could turn herfelf in bed, and lie on either fide, and not only move her legs, but walk much better. She frequently after this had flight fhivering fits, and a fenfe of coldnefs down her back, followed by reftleffnefs and feverifh heat, efpecially in her hands and feet in the evening, which went off with a free perfpiration towards morning. Her pulfe was at all times very quick.

Though one or more ftools had been regularly procured every day, an immenfe quantity of hardened fæces, of a large volume, were now difcharged for three or four fucceffive days, by which her fize was much leffened. She was foon after able to bear a journey to *London*, her friends being folicitous that the nature of her complaint fhould be afcertained, as there had been various opinions and reprefentations made of it, by different gentlemen who had feen her in the country.

On Sunday, *March* 31ft, I vifited this lady, and as it feemed of principal importance to difcover in the firft place, the feat and nature of her difeafe, it was neceffary to be particular in my inquiries and examination. The whole *abdomen* was diftended by a circumfcribed tumour, evidently connected with, and fpringing from the right fide, near the groin, thence extending acrofs, and high up in the *abdomen*. This tumour, though not perfectly uniform over its furface, was diftinctly circumfcribed, and I thought I could perceive an obfcure fluctuation in it. I could alfo feel an angle of the tumour in the pofterior part of the *pelvis*, by which the *os uteri* was projected fo high, and fo far forwards, as to be almoft beyond my reach, as is the cafe in a retroverfion of the *uterus*. I could alfo afcertain that fhe was not pregnant. I did not therefore hefitate to give my opinion, that it was a *dropfy of the ovarium;* and by fuppofing this, early in the difeafe, to have dropped low down in the *pelvis*, and, afterwards to have rifen according to its increafe, all the fymptoms which had occured in the courfe of the difeafe, could be fatisfactorily explained.

Having reprefented my opinion to the patient and her friends, though I could give but little hope of the difeafe being cured, I freed them from the fear and folicitude of any immediate danger.

The

The following draught was the only medicine I advised.

R/ Flor. Chamæmel. pulv. gr. xv.

Rad. Rhei pulv. gr. v

—— Zingiber. pulv. gr. iij.

Aqu. Ment. sativ. unc. ij m. f. Haustus

Sumat ter quottidie.

On the following day, she informed me that after suffering considerable pain in the bowels, she had had four or five copious motions, and that after every motion, she was sensible of her size decreasing. The motions were unusually offensive, and, before they came away, the desire to expel them was unnaturally urgent and painful. On examining them, I found that they almost wholly consisted of a gelatinous fluid, with many streaks of blood, and with little or no mixture of fæces.

The same medicines were repeated.

On *Tuesday*, after several other motions of the same kind, the distention of the *abdomen* was lessened more than one half, and instead of feeling weakened by the evacuations, the patient felt herself very much relieved, and cheered with the prospect of a speedy recovery. She took a sufficient quantity of nourishment, and continued the same medicine.

On *Wednesday*, I had nearly the same account of the number of motions, and of the gradual decrease of the swelling of the *abdomen*, which was now in fact wholly gone, except that I could feel the small tumour formed by the cyst, in which the fluid had been contained.

On examining this day *per vaginam*, the *os uteri* was found to be descended into its proper situation, and no tumour whatever remained in the cavity of the *pelvis*. The patient in short felt, and considered herself as well, in which sentiment I encouraged her; concluding in my own mind, that, in consequence of preceding inflammation, an adhesion had taken place between the cyst of the tumour, and some part of the intestine, probably the *rectum*, the adhering portion of the bowel had given way, and, by that opening, the contents of the tumour had been evacuated.

At my request this patient stayed in town for a month, at the end of which time I saw, and examined her again; but I should not then, either from the state of her health, or any thing I could discover, have suspected her ever to have suffered from any such complaint as that I have been describing.

Old Burlington-Street,
*July* 6, 1799.

